# Preparation and Characterization of Selenium Incorporated Guar Gum Nanoparticle and Its Interaction with H9c2 Cells

**DOI:** 10.1371/journal.pone.0074411

**Published:** 2013-09-30

**Authors:** Rema Sreenivasan Soumya, Vadavanath Prabhakaran Vineetha, Premachandran Latha Reshma, Kozhiparambil Gopalan Raghu

**Affiliations:** Agroprocessing and Natural Products Division, CSIR- National Institute for Interdisciplinary Science and Technology (NIIST), Thiruvananthapuram, Kerala, India; RMIT University, Australia

## Abstract

This study deals with the preparation and characterization of selenium incorporated guar gum nanoparticle (SGG), and its effect on H9c2 cardiomyoblast. Herein, nanoprecipitation techniques had been employed for the preparation of SGG nanoparticle. The prepared nanoparticle had been subjected to various types of analytical techniques like transmission electron microscopy (TEM), X-ray diffraction (XRD) and particle size analysis to confirm the characteristics of nanoparticle as well as for selenium incorporation. Physical characterization of nanoparticle showed that the size of nanoparticles increase upto ∼69–173 nm upon selenium incorporation from ∼41–132 nm. Then the prepared nanoparticles were evaluated for its effect on H9c2 cells. In this regard, the effect of nanoparticle on various vital parameters of H9c2 cells was studied. Parameters like cell viability, uptake of selenium incorporated guar gum nanoparticle by the cells, effect of SGG on DNA integrity, apoptosis, reactive oxygen species generation, alteration in transmembrane potential of mitochondria and cytoskeletal integrity had been investigated. Viability results showed that up to 25 nM of SGG was safe (10.31%) but beyond that it induces cytotoxicity. Cellular uptake of selenium showed that cell permeability for SGG is significantly high compared to normal selenium (7.2 nM of selenium for 25 nM SGG compared with 5.2 nM selenium for 25 nM sodium selenite). There was no apoptosis with SGG and also it protects DNA from hydroxyl radical induced breakage. Likewise no adverse effect on mitochondria and cytoskeleton was observed for 25 nM of SGG. Overall results reveal that SGG is highly suitable for biomedical research application.

## Introduction

Nanoscience has become an important area of research in biomedical sciences. Nanoparticles deliver a wide range of drugs (hydrophilic drugs, hydrophobic drugs, proteins, vaccines, biological macromolecules) to target areas of the body (lymphatic system, brain, arterial walls, lungs, liver, spleen, or made for long-term systemic circulation) for sustained periods of time [1]. Currently different materials are under study for their suitability for enhanced drug delivery in various disorders. In recent years, a large number of studies have been conducted on polysaccharides and their derivatives for their potential application as drug delivery systems [2]–[4]. Polysaccharides have a large number of reactive groups, with wide range of molecular weight and they vary in their chemical composition which contributes to their diversity in structure and property. As natural biomaterials, polysaccharides are highly stable, safe, nontoxic, hydrophilic and biodegradable. Nanoprecipitation is a general route to prepare polymeric nanoparticles under mild conditions and is well suitable for biological applications [5], [6]. These techniques have many advantages as it is a straightforward technique, rapid and easy to perform [7]–[9]. This method does not require extended shearing/stirring rates, sonication or very high temperatures, and is characterized by the absence of oily-aqueous interfaces, all conditions that might damage a protein structure. Moreover, surfactants are not always needed and toxic organic solvents are generally excluded from this procedure. Nanoparticles made of biodegradable polymers like proteins and polysaccharides can act as efficient drug delivery vehicles for sustained, controlled and targeted release, aiming to improve the therapeutic effects and also to reduce the side effects of the formulated drugs [10].

Guar gum (GG) is a water soluble polysaccharide derived from the seeds of *Cyamopsis tetragonolobus*, family of Leguminosae. It consists of linear chains of (1→4) –β- D- mannopyranosyl units with α-D-galactopyranosyl units attached by (1→6) linkages [11]. In pharmaceutical formulations, GG is used as a binder, disintegrant, suspending and thickening agent, and stabilizing agent. Soluble polysaccharides like GG have been explored as possible ingredients in the development of “functional foods” because of their ability to reduce plasma cholesterol and consequently contribute to the reduction of the risk of cardiovascular disease [11]. GG and its derivatives in various forms such as coatings, matrix tablets, hydrogels and nano/microparticles can be exploited as potential carriers for targeted drug delivery [12]. Most of the natural polysaccharides have hydrophilic groups such as hydroxyl, carboxyl and amino groups, which could form non-covalent bonds with biological tissues (mainly epithelia and mucous membranes), forming bioadhesion [13]. There is not much information available in the literature on guar gum based nanoparticle for biomedical application and this is the novel approach in this regard.

Selenium (Se) is an essential mineral for optimal health. It is a structural component of the active centre of many antioxidant enzymes and functional proteins. Thus, cellular Se status plays an important role in the reduction of oxidative stress in the body [14]. It plays an important role to prevent various diseases, such as diabetes, hypercholesterolemia [15], cardiovascular disease [16], [17] and certain cancers [18]. But excessive Se intake can result in adverse health effects. Therefore, Se has either nutritional function or toxicity depending on its concentration and species. Selenium mainly consists of two inorganic forms, selenite and selenate. It has been also evidenced that due to its prooxidant property supplementation of selenite to mice and cells may cause various adverse effects, which depends on the concentration and other factors [19]–[23]. Therefore, how to provide efficient and safe application of dietary selenite supplementation has become a challenge topic in recent years.

In the present study, GG was selected as a carrier due to its widespread use as fiber for reducing blood cholesterol level, obesity and hyperglycemia and Se was the therapeutic agent. We followed nanoprecipitation method for the preparation of GG nanoparticle and selenium incorporated guar gum nanoparticle (SGG). The physicochemical properties of the nanoparticles were characterized by particle size analyzer, TEM and XRD. In order to see the interaction of SGG with cells we systematically investigated the effect of nanoparticle on H9c2 cells by analyzing various parameters like cell viability, apoptosis, DNA protection, reactive oxygen species (ROS) generation, mitochondrial transmembrane potential change and alteration in cytoskeleton.

**Table 1:** Viability of H9c2 cardiac myoblast cells treated with sodium selenite and SGG nanoparticles.

	Concentration (nM)	% toxicity		
		1 h	6 h	24 h
Sodium selenite	5	1.73±0.39	5.41±0.87	11.39±1.84
	25	2.38±0.43	6.18±0.63	13.66±1.07
	50	9.05±0.48	11.36±0.87	14.59±1.12
GG nanoparticles	5	2.63±0.37	6.50±0.87	10.33±1.72
	25	4.44±0.89	7.43±1.00	13.59±0.99
	50	5.14±0.30	9.34±0.89	14.60 ± 1.47
SGG nanoparticles	5	1.79±0.64	3.38±1.03	7.21±1.23
	25	2.61±0.93	6.30±0.80	10.31±2.32
	50	10.87±1.12	12.54±0.67	17.40±1.51

## Materials and Methods

### Materials and Reagents

Guar gum powder, mannanase enzyme from *Helix pomatia*, sodium selenite, triton X-100, isopropanol, 3-(4,5-dimethylthiazol-2-yl)-2,5-diphenyl tetrazolium bromide (MTT), sodium tripolyphosphate, dimethyl sulfoxide (DMSO), 2′,7′ dichlorodihydrofluorescein diacetate (DCFH-DA), acridine orange (AO), ethidium bromide (EtBr), 2, 3 diaminonaphthalene, JC-1 (5,5′,6,6′-tetrachloro-1,1′,3,3′tetraethylbenzimidazolyl carbocyanine iodide), 4′,6-diamidino-2-phenylindole (DAPI), phallodin and pUC-18 plasmid DNA were purchased from Sigma Chemicals, USA. Dulbecco's modified Eagle's medium (DMEM), foetal bovine serum (FBS) and supplements were from Himedia Pvt Ltd India.

### Preparation and Characterization of GG nanoparticle and SGG

The GG nanoparticle was prepared by nanoprecipitation method [24]. Briefly 1% GG was depolymerised with mannanase enzyme with a pH of 5.2 in citrate phosphate buffer and incubated at 30±2°C for 24 h. The hydrolysed suspension of GG was filtered through 0.2 µm syringe filters. The filtered suspension was stored in 4°C for further characterization. Then through same nanoprecipitation method SGG were prepared with depolymerised GG, sodium selenite, Triton X 100, isopropanol and sodium tripolyphosphate. The solution was vortexed and sonicated for 10 min at room temperature, filtered with 0.2 µm syringe filters to get uniform nanoparticles.

The average particle size (hydrodynamic diameter, Zaverage) of the prepared particles (GG, SGG) were determined by photon correlation spectroscopy (PCS) using 3000 HSA Zetasizer (UK) equipped with He–Ne laser ( = 633 nm). In addition morphology and particle size of the samples were analyzed using high resolution FEI Tecnai transmission electron microscope, Netherlands. XRD of GG, pure sodium selenite and SGG were measured using X-ray diffractometer (XPERT, Philips, Eindhoven, Netherlands) with nickel-filtered Cu-K radiation ( = 0.154 nm) at a voltage of 40 kV and a current of 30 mA.

### Cell Culture

Rat embryonic cardiomyoblast derived H9c2 cells were obtained from National Centre for Cell Science (NCCS), Pune, India. H9c2 cells mimic most of the characteristic features of adult cardiac myocytes and this is an ideal cell line to check the effect of drug on myocardium in cell culture system. Cells were cultured in DMEM supplemented with FBS (100 U penicillin/mL), and 100 μg streptomycin/mL and cultured in 5% CO_2_ at 37°C. Cells were passaged regularly and subcultured to 80% confluence before the experiments. Fresh nanoparticle solutions were prepared and checked to ensure consistency of physical and chemical properties of nanoparticles. Nanoparticles were freshly prepared before use and vortexed thoroughly before being added to the cells. Experimental design consist of following groups unless otherwise specified (1) control cells (2) cells treated with sodium selenite alone (5, 25 and 50 nM) and (3) cells treated with SGG (5, 25 and 50 nM). Observations were made after 1, 6 and 24 h of incubation.

### Evaluation of Cell Viability

Cell viability was determined by MTT assay. Cells in exponential growth phase were plated at 5×10^4^ cells per well in 24-well plate. After, cells were exposed to various concentrations (5, 25 and 50 nM) of sodium selenite, and SGG for 1, 6 and 24 h, they were subjected to MTT analysis. For this 350 μL of MTT solution (5 mg/mL) was added to each well and incubated for 4 h at 37°C. The formazan crystals thus formed were dissolved in DMSO. Then the plates were read after 45 min in a microplate reader (Biotek Synergy 4, US) at 570 nm) and percentage of viable cells were calculated.

### Intracellular Localization of SGG nanoparticles

Initial experiments were conducted to see whether H9c2 cells uptake nanoparticles. We found that both GG nanoparticle and SGG emit autofluorescence at blue region (excitation: 360 nm; emission: 435 nm). For cellular uptake studies, cells were seeded in 96-well plate at a density of 5×10^3^ per well and treated with various concentrations of SGG (5, 25 and 50 nM) for 1, 6 and 24 h and single concentration (50 nM) of macro guar gum (MGG) as reference. The cells of all experimental groups were subjected to DNA staining with AO (exicitation: 502 nm; emission: 525 nm). Then the cells were subjected to fluorescent imaging by spinning disk microscopy (BD Pathway™ Bioimager system, USA).

### Estimation of Intracellular Se concentration

Cells were treated with various concentrations of Se and SGG (5, 25 and 50 nM) for 24 h to check the influx of Se. For this 100 µl of cell homogenate were digested with 500 µl of HNO_3_/HClO_4_ (4∶1; v/v) at 190°C for 90 min. After cooling to room temperature, 500 µl of 5 mol/L HCl was added and the open glass tubes were heated to 150°C for 30 min. Then 2 ml of 2.5 mM EDTA and 500 µl diaminonaphthalene reagent was added at room temperature and the mixture were left at 55°C for 30 min. 1 ml portion of cyclohexane was used to extract the piazselenol and fluorescence was measured using a fluorimeter at excitation of 364 nm and emission of 520 nm.

### Nanoparticle Interaction on Plasmid DNA

To check whether nanoparticle prepared has any effect on DNA we conducted experiment on plasmid pUC 18. The reaction mixture consisting of plasmid DNA and various concentrations of SGG was subjected to agarose gel electrophoresis and visualized by EtBr staining. In addition, we also conducted study to see whether SGG protect DNA from hydroxyl radical induced damage. For this, the reaction was conducted at a total volume of 14 µl containing 2 µl of plasmid pUC 18 DNA (50 ng DNA/µl) in 5 µl of 5, 25 and 50 nM concentration of Se and SGG, 7 µl of Fenton's reagent. The DNA (supercoiled, linear and open circular) was analyzed on 1% agarose gels and visualized. Ellagic acid was used as positive control.

### DNA Integrity

Briefly the cells in all experimental groups were stained with AO (excitation: 502 nm; emission: 525 nm) and EtBr (excitation: 510 nm; emission: 595 nm) to detect apoptosis and processed for fluorescent imaging to see alteration with various treatments. The working stain (100 µg/mL of AO and 100 µg/mL EtBr in phosphate buffered saline) was added to cells and then examined under spinning disc fluorescent microscope.

### Reactive Oxygen Species Generation with Nanoparticles

Oxidative stress in response to nanoparticle incubation was measured by determining intracellular ROS generation. Intracellular ROS was determined by oxidative conversion of cell-permeable DCFH-DA to fluorescent 2′, 7′ dichlorofluorescein (DCF). For this H9c2 cells were seeded in 96-well plate at a density of 5×10^3^ cells per well and treated with different concentration of Se and SGG (5, 25, 50 nM) with different time duration. DCFH-DA stain in serum free medium was co-incubated with H9c2 cells at 37°C for 20 min. After three washes, DCF fluorescence was measured by fluorimetry (excitation: 488 nm; emission: 525 nm) in multiwell plate reader and fluorescent imaging was done to detect the difference in the intensity of fluorescence emitted.

### Alteration in Mitochondrial Membrane Potential (ΔΨm)

The cells were seeded in 96-well plate at a density of 5×10^3^ cells per well in 200 µL of culture medium and treated with different concentration of Se and SGG (5,25 and 50 nM) for 24 h. The experiment was done as per the protocol provided with the kit (JC1 kit, Sigma). In normal cells, the JC-1 dye concentrates in the mitochondrial matrix, where it forms red fluorescent aggregates because of the electrochemical potential gradient. Dissipation of ΔΨm prevents the accumulation of JC-1 in the mitochondria and thus it is dispersed throughout the cell, leading to a shift from red (J-aggregates) to green fluorescence (JC-1 monomers) and visualized under spinning disk microscope and fluorescence intensity was measured in multiwell plate reader. For JC-1 monomers, the fluorimeter was set at 490 nm excitation and 530 nm emission wavelengths, and for J- aggregates, the fluorimeter was set at 525 nm excitation and 590 nm emission wavelengths. Valinomycin (1 mg/mL) was used as positive control.

### Cytoskeleton Integrity

The cells from experimental groups were washed with PBS. Then cells were fixed with 4% paraformaldehyde in PBS for 10 min, permeabilized and dehydrated with cold 100% acetone for 3–5 min. Phalloidin stain (excitation: 488 nm; emission: 525 nm) was added and kept at room temperature for 30 min. Nucleus was counterstained with DAPI (excitation: 360 nm; emission: 450 nm) and visualized [25].

### Statistical Analysis

All experiments were performed in sextuplicates (n = 6). Data were reported as mean ± SD. The data were subjected to two-way analysis of variance (ANOVA) and the significance of differences between means were calculated by Duncan's multiple range test using SPSS for windows, standard version 7.5.1, and the significance accepted at P≤0.05.

## Results and Discussion

In the recent years, a wide range of nanomaterials have been developed for biomedical applications due to their unique properties. The special physicochemical properties of nanomaterial make them have entirely different effect on biological system compared to their macro counterparts. Therefore, the biological properties of nanomaterial were studied for its possible biomedical applications. Nanoparticles containing metals of biological importance are attracting much attention of present scenario because of their physical and chemical properties. Taking this into account, the present study was aimed to prepare and characterize SGG and study its interaction with H9c2 by assessing its effect on various vital parameters. For this we conducted batteries of *in vitro* experiments to evaluate its interaction with H9c2 cell lines.

### Particle Size Measurements

**Figure 1:**
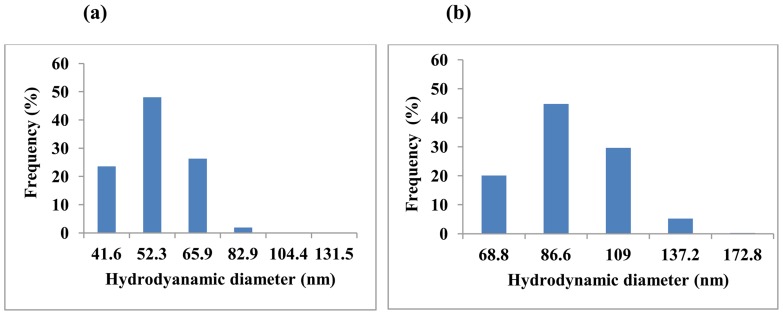
Particle size distribution of nanoparticle. Nanoparticles were prepared by nanoprecipitation method. The prepared nanoparticles were subjected to particle size analysis. (a) GG nanoparticle had an average particle size varies from 41 to 132 nm range and (b) SGG nanoparticle prepared by nanoprecipitation method shows an increase in particle size from 69 to 173 nm.

In the present study GG nanoparticles were prepared by nanoprecipitation method. Particle size analysed by PCS indicated the presence of fine spherical nanoparticles of size of ∼41–132 nm with a polydispersity index (PDI) of 0.4 (Fig. 1a). The size of the nanoparticle had increased to ∼69–173 nm range upon Se incorporation (Fig. 1b). This result is expected since selenite carried negative charges and electrostatically interacted with guar gum, which would promote formation of nanoparticles through ionic cross-linking. Particle size is one of the most important parameters determining biocompatibilities and bioactivities of materials of therapeutic importance.

### Transmission Electron microscopic Images of GG nanoparticle and SGG

**Figure 2:**
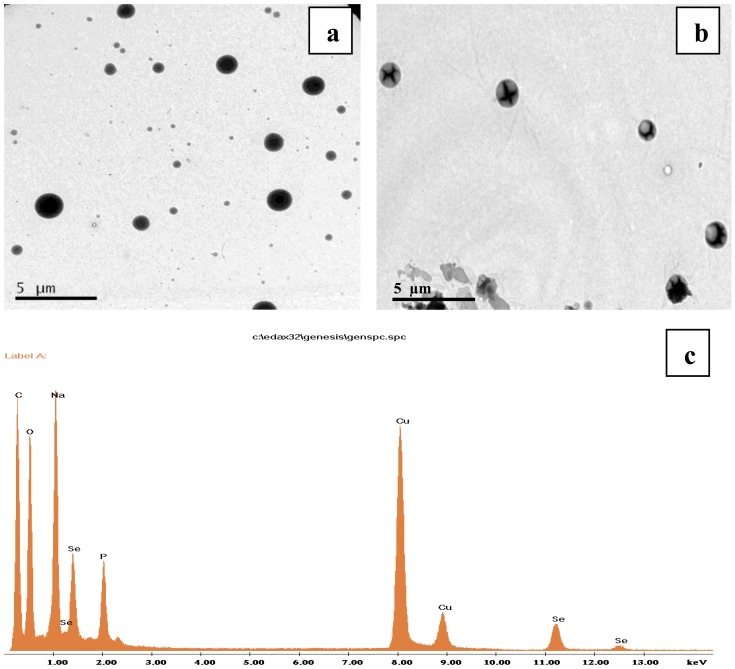
TEM images of (a) GG showing spherical morphology with a size of ∼40 nm with few larger particles (b) SGG nanoparticles were also spherical in shape, upon incorporation of selenium the particle size has been increased to the range of 50–100 nm (c) EDX spectrum of SGG show the presence of Se peaks, thereby confirming the presence of Se in SGG.

The TEM analysis of GG nanoparticle revealed the presence of fine spherical nanoparticle of size ∼40 nm range with few larger particles. It was found that the sizes observed by DLS were larger than those determined by TEM images. This might be due to the fact that GG binds to the surface of the selenium which in turn creates a layer and this has made the particles appear larger. TEM images also confirmed that upon Se incorporation the size of GG nanoparticle had increased to ∼50–100 nm (Fig. 2a, b). An elemental composition analysis by TEM-EDX showed the presence of strong signals from the Se atoms, together with signal of C, Na and O atom from SGG (Fig. 2c) confirming the presence of Se in SGG.

### XRD Characterization of Nanoparticles

**Figure 3:**
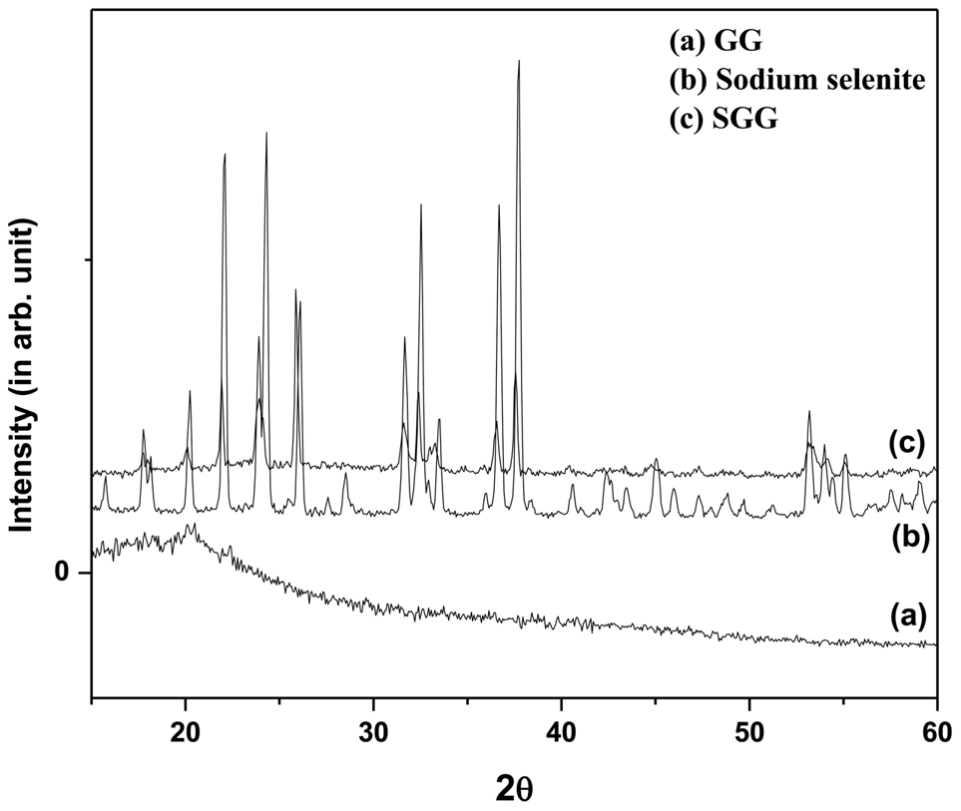
XRD images of (a) GG (b) sodium selenite and (c) SGG nanoparticle. GG nanoparticle appears amorphous in nature in their morphology as per XRD data (Fig. 3a). While sodium selenite appears crystalline nature in the XRD pattern (Fig. b) The presence of major peaks of selenium in SGG confirm the incorporation of selenium in GG nanoparticle (Fig. 3c).

In nanoparticle preparation it is very important to control the particle size, shape and morphology. The SGG was characterized by XRD as it is an important analysis tool in nanomaterial science. Figure 3a, b, c show the powder X- ray diffraction patterns of GG, sodium selenite and SGG respectively. XRD of GG (Fig. 3a) was amorphous because of its polymeric nature. XRD pattern of pure sodium selenite is shown in figure 3b. Peaks corresponding to sodium selenite had been observed in figure 3c, confirming the incorporation of Se in SGG.

### Morphological Analysis of Cells upon Treatment with Nanoparticles

**Figure 4:**
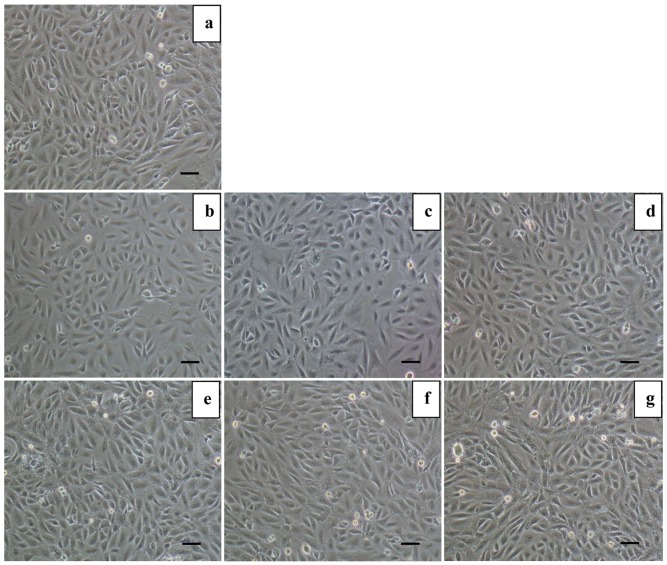
Morphological examination of cells with nanoparticles. Images of H9c2 cells from different experimental groups under phase-contrast microscope (10x). (a) Control cells; (b, c, d) Cells treated with 5, 25 and 50 nM Se respectively; (e, f, g) Cells treated with 5, 25 and 50 nM SGG, respectively. H9c2 cells showed no significant alteration in morphology upon treatment with Se and SGG of any concentrations reported in the study. Scale bar corresponds to 100 µm.

The cytotoxicity of the SGG nanoparticle was verified for its biomedical application. H9c2 myoblast are spindle to stellate shaped that can be mono or multinucleated. The morphological examination of the cells treated with different doses of both SGG and Se showed normal cell morphology up to 50 nM for 24 h exposure (Fig. 4).

### MTT Assay

Nanomaterials are used increasingly in food, cosmetics, medical imaging, disease diagnosis and drug delivery. However more and more evidence indicates that reduction to nanoscale causes marked differences in properties compared with macroscale. In order to evaluate the cytotoxicity of the newly synthesized SGG and the bulk Se, H9c2 cells were treated with various concentrations (5, 25, 50 nM) for various duration (1, 6 and 24 h). The results showed that 5 and 25 nM of Se and SGG nanoparticle for 1, 6 and 24 h of incubation were non-toxic. But higher dose (50 nM) of Se and SGG showed significant toxicity of 17.4% and 14.59% after 24 h incubation respectively (Table.1).

### Uptake of SGG by H9c2 cell

An important factor that usually contributes to nanomaterial based drug cytotoxicity is cellular uptake [26]. GG is a nonionic polysaccharide that is abundantly present in nature and has many properties desirable for drug delivery applications. Due to the presence of various functional groups on molecular chains polysaccharides can be easily modified chemically and biochemically, resulting in different kind of derivatives [27]. In addition they are highly stable, safe, non toxic, hydrophilic and biodegradable. Moreover the cost of processing is very low. Among polysaccharides GG is a potential candidate for drug delivery application due to its drug release retarding property and susceptibility to microbial degradation in the large intestine [28]. It is established fact that nanoparticles can efficiently intrude in cell by exploiting endocytosis machinery. But only specialised cells such as macrophages are capable of phagocytosis. On the other hand almost all cells internalize nanoparticles by pinocytosis. There are many factors like physicochemical properties like size, shape, surface charge and surface chemistry that has been identified for modulating cellular uptake efficiency.

**Figure 5:**
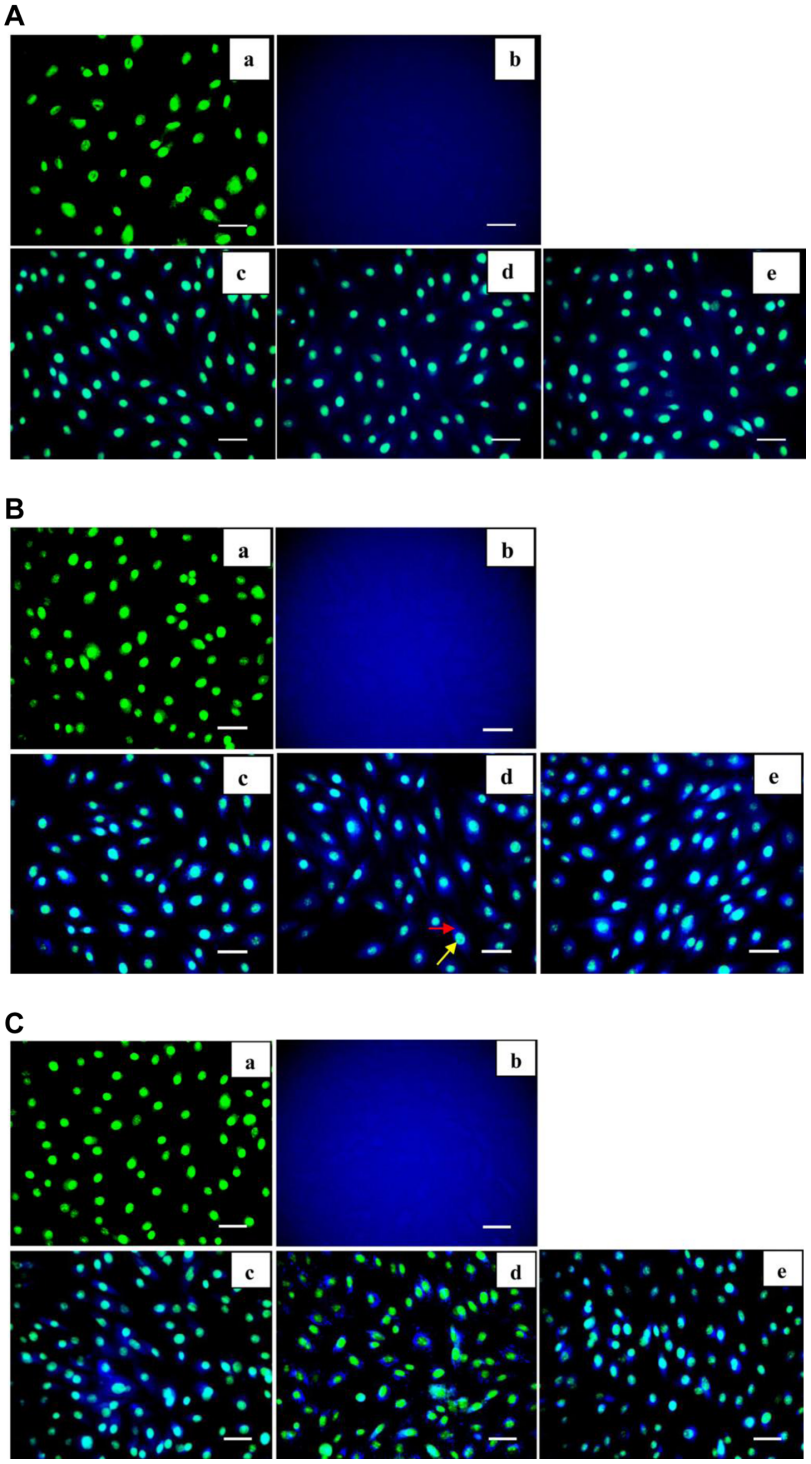
A. Uptake of SGG by H9c2 cardiac myoblasts after 1(a) Control; (b) MGG; (c, d, e) Cells treated with 5, 25 and 50 nM of SGG. The autofluorescent property of guar gum was utilized for the uptake study. The cells were counter stained with AO that binds with DNA of live cells to distinguish nucleus from cytoplasm. The green colour represents nucleus and blue colour is the autofluorescence emitted by GG. Figure 5b appears like blue cloud due to the autofluorescence of MGG in the medium. The presence of blue fluorescence (Fig. 5c, d, e) in the cytoplasm of cells clearly reveals the uptake of SGG by the cells in a dose dependent manner. Scale bar corresponds to 100 µm. **B. Uptake of SGG by H9c2 cardiac myoblasts after 6 h incubation** (a) Control; (b) MGG; (c, d, e) Cells treated with 5, 25 and 50 nM SGG. The intensity of fluorescence is more in these figures compared to 5A (c, d, e) revealing the duration dependent uptake of SGG by cells. Red arrow points cytoplasm and yellow arrow point nucleus respectively. Scale bar corresponds to 100 µm. **C. Uptake of SGG by H9c2 cardiac myoblasts after 24 h incubation** (a) Control; (b) MGG; (c, d, e) Cells treated with 5, 25 and 50 nM SGG. Fluorescent intensity of the cells in this group was high compared to the other 2 groups (Figs. 5B c, d, e) confirming the duration dependent uptake of SGG by cells. Scale bar corresponds to 100 µm.

To evaluate the cellular uptake of SGG and MGG we utilized autofluorescence property of GG. For this, cells were incubated with various concentrations (5, 25 and 50 nM) of SGG and MGG (50 nM) for 1, 6 and 24 h, and counter stained with AO that stains the double and single stranded DNA of live cells that appears green in color and were subjected to fluorescence imaging by spinning disk microscopy. We observed presence of fluorescence in the SGG treated cells (Fig. 5A, 5B and 5C) whereas no fluorescent emission was observed in MGG treated cells (Fig. 5A (b), 5B (b), 5C (c)). This observation reveals the presence of SGG in cells not MGG. In addition, fluorescent data showed uptake of nanoparticle was dose and duration dependent. The maximal cellular uptake was found in 24 h exposure with high dose (Fig. 5C).

### Differential Se Uptake by H9c2 cells

**Figure 6:**
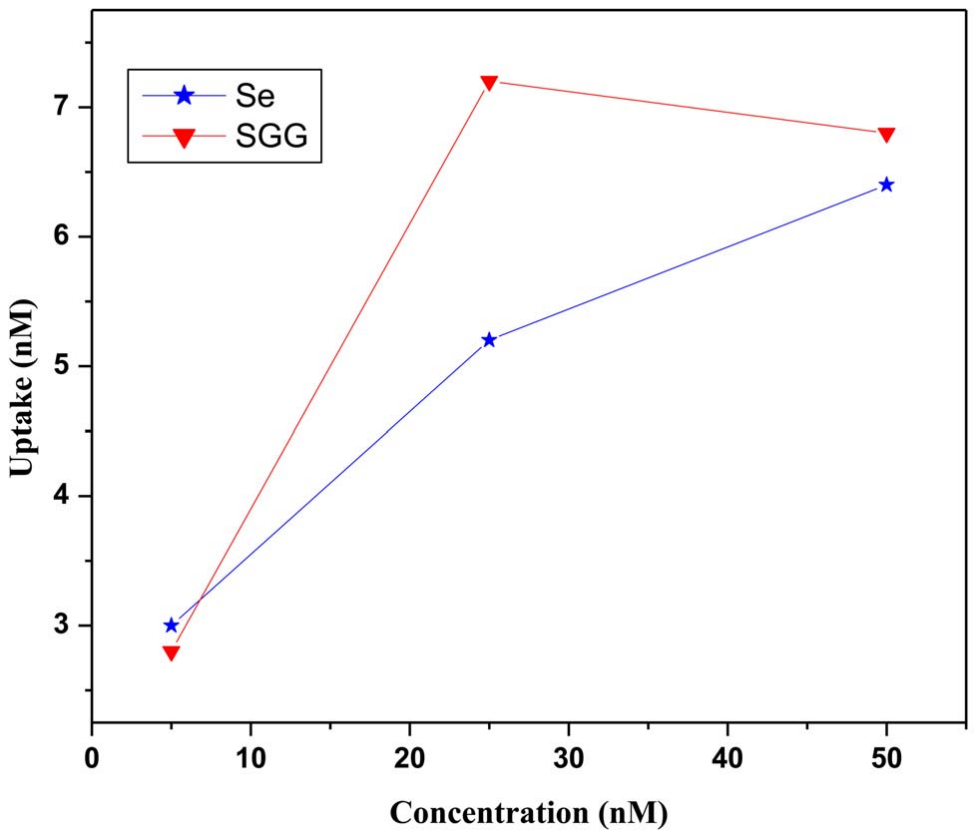
*In vitro* cellular uptake of Se from sodium selenite and SGG. Se uptake study using diaminonaphthalene method shows that uptake of Se by cell is higher for SGG (red) than sodium selenite (blue). 25 nM of both Se and SGG showed maximum uptake of Se by cells.

Se is a trace element with wide commercial applications, due to its special chemical and physical properties. Se nanoparticles attracted more attention in the past decade due to their high bioavailability and antioxidant activities, comparative low toxicity and novel therapeutic property [29]–[32]. We examined the cellular uptake of Se from sodium selenite and SGG in H9c2 cell lines with diaminonaphthalene reagent. For this, cells were treated with different concentrations of sodium selenite (5, 25 and 50 nM) and SGG (5, 25 and 50 nM) for 24 h. Analysis showed that 25 nM of SGG was found to have more effective uptake of Se (7.2 nM) which was found to be significantly greater than Se uptake from sodium selenite (5.2 nM). Beyond 25 nM, concentration did not show any influence on Se uptake whether in SGG or sodium selenite (Fig. 6). The more cell permeability of nanoparticle will enable to have more drug bioavailability at target site for better therapeutic property. This also reduces the dose of drug required for recovery and reduces the adverse effect of drug. There are reports to suggest that nanoencapsulation of Se in polysaccharide like chitosan increase cellular Se level [33]. Se in encapsulated stage desensitizes the cells to Se compounds and decrease damage to cells in contrast to application of native Se to the cells [33]. This explains why more Se in guar gum encapsulated form is taken up by the cells. Another reason for the minimum toxicity with SGG is due to the fact that SGG are not metabolically available for the induction of any type of cell damage.

### Effects of SGG on Apoptosis

**Figure 7:**
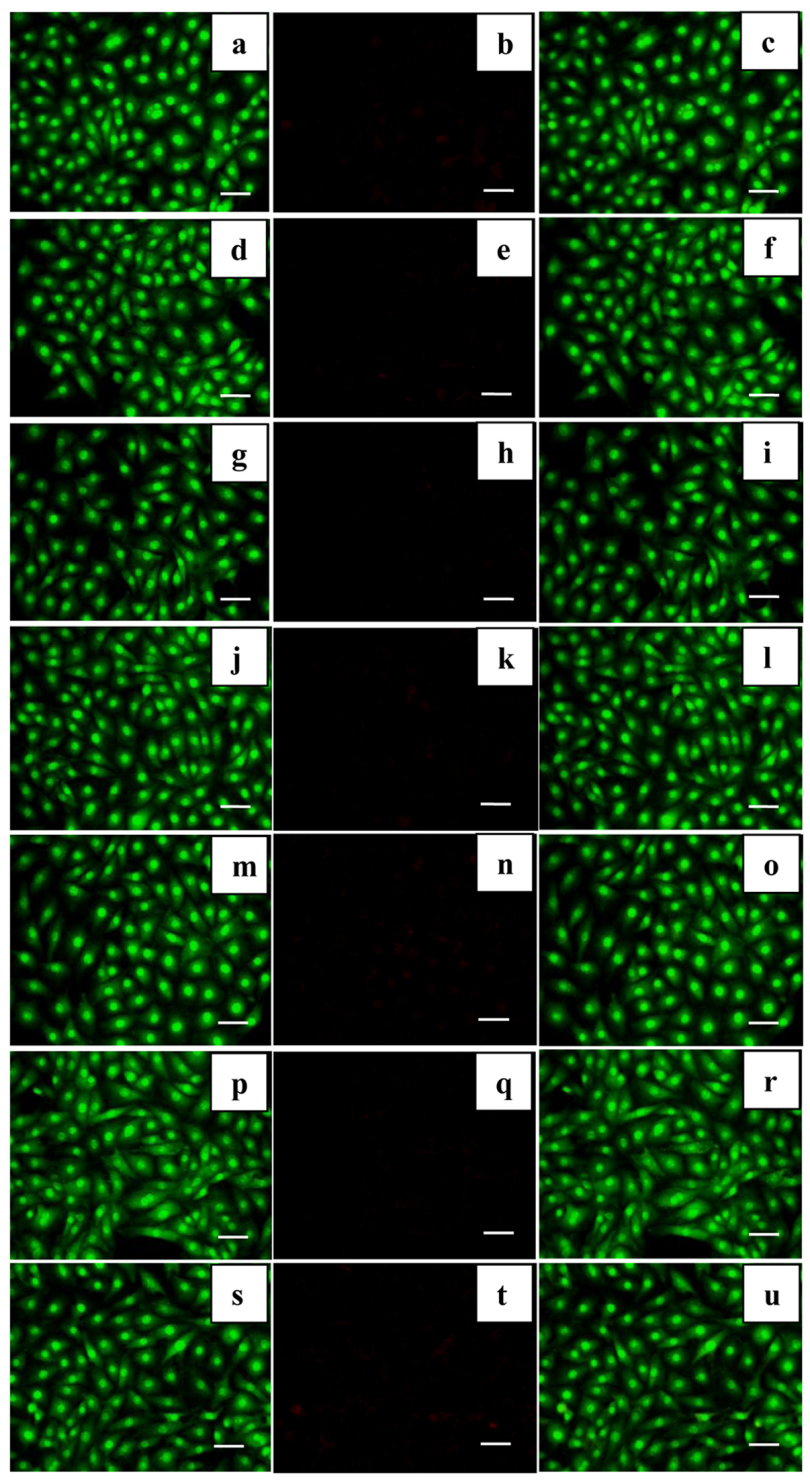
Alteration in DNA integrity with Se and SGG nanoparticle. Photomicrographs of H9c2 cells with AO/EtBr staining to check induction of apoptosis with Se and SGG (20×); (a) Control cells stained with AO; (b) Control cells stained with EtBr; (c) superimposed image of a and b (d) H9c2 cells treated with 5 nM Se stained with AO; (e)H9c2 cells treated with 5 nM Se stained with EtBr; (f) Superimposed images of d and e; (g) H9c2 cells treated with 25 nM Se stained with AO; (h) H9c2 cells treated with 25 nM Se stained with EtBr; (i) Superimposed images of g and h; (j) H9c2 cells treated with 50 nM Se stained with AO; (k) H9c2 cells treated with 50 nM Se stained with EtBr; (l) Superimposed images of j and k respectively; (m) H9c2 cells treated with 5 nM SGG stained with AO; (n) H9c2 cells treated with 5 nM SGG stained with EtBr; (o) Superimposed images of m and n; (p) H9c2 cells treated with 25 nM SGG stained with AO; (q) H9c2 cells treated with 25 nM SGG stained with EtBr; (r) Superimposed images of p and q (s) H9c2 cells treated with 50 nM SGG stained with AO; (t) H9c2 cells treated with 50 nM SGG stained with EtBr; (u) Superimposed images of s and t. The green color represents viable cells stained with AO. This reveals clearly that there was no induction of apoptosis with Se or SGG. Scale bar corresponds to 100 µm.

In order to characterize the safety profile of the SGG we investigated whether SGG induce apoptosis in H9c2. Induction of apoptosis is considered an important cellular event that can account for the cancer preventive effects of Se compounds. Apoptosis induced by supranutritional doses of seleno compounds are described in various types of neoplastic cells, including prostate, colon and liver cancer, leukemia and lymphoma. Se in encapsulated form at nanoparticle size upregulates selenoenzyme [34]. To evaluate apoptosis inducing property of SGG, cells were stained with AO/EtBr and it was found that the exposure of H9c2 cells with different concentrations of 5, 25 and 50 nM of Se and SGG nanoparticle for 24 h did not cause apoptosis (Fig. 7). SGG was effective to protect the cells from apoptosis at 50 nM for 24 h and this property of SGG will definitely help us to use this nanoparticle for therapeutic purpose against various disorders.

### DNA Protection Assay

**Figure 8:**
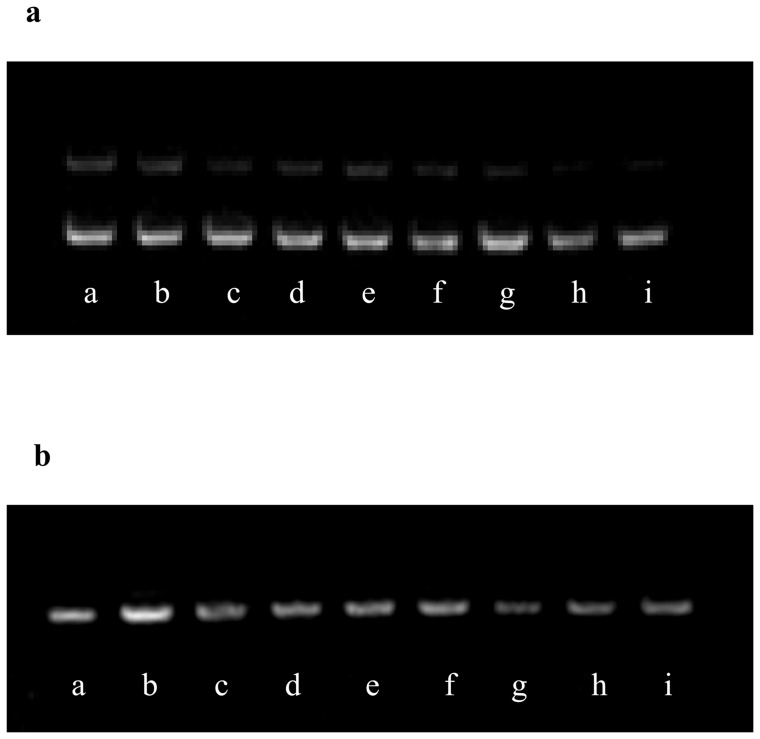
A. DNA damage protection by SGG. Modulating effect of SGG against Fentons reaction induced plasmid conformational change. Figure 8A shows gel view of plasmid DNA clevage assay, OC and SC indicate the open circular and supercoiled plasmid forms respectively. (a) Control DNA pUC18 plasmid alone, (b) the plasmid DNA pUC18 was incubated with Fentons reagent, (c) with ellagic acid 50 nM, with different concentrations of Se (d) 5 nM (e) 25 nM (f) 50 nM and different concentration of SGG (g) 5 nM (h) 25 nM (i) 50 nM. SGG nanoparticle effectively protects the DNA from plasmid breakage in a dose dependent manner. **B. The effect of nanoparticle on DNA.** This gel view shows plasmid conformation with various doses of Se and SGG revealing clearly the non toxicity of the particle on plasmid. (a) Control DNA pUC18 plasmid alone, (b, c) with ellagic acid 5 nM and 50 nM, with different concentrations of Se (d) 5 nM (e) 25 nM (f) 50 nM and different concentration of SGG (g) 5 nM (h) 25 nM (i) 50 nM.

Oxidative DNA damage has been implicated in various degenerative diseases. Sodium selenite is reported to inhibit oxidative DNA damage caused by iron (Fe^2+^) in the presence of H_2_O_2_, in a cell free system, which contained plasmid DNA, Fe^2+^ and H_2_O_2_ [35]. The effect of GG nanoparticles and SGG on Fe^2+^ dependent hydroxyl radical induced DNA damage of pUC18 plasmid was studied. The treatment of super coiled (SC) DNA with Fenton's reagent directed to the alteration of DNA to open circular form (OC). The addition of SGG nanoparticles to the reaction mixture substantially decreased the DNA strand scission and retained the SC form, thus effectively protect DNA, in a dose dependent manner (Fig. 8A). In addition we also checked whether it induce breakage or nick or ladder formation on plasmid DNA (pUC18) by agarose gel electrophoresis (Fig. 8B). It did not affect plasmid DNA. This DNA protection property of SGG will have therapeutic potential as in some metabolic disorders like diabetes, hydroxyl radical induced DNA damages are very common. From these results it is again clear that SGG prepared by nanoprecipitation is safe as well as it has therapeutic potential to use for biomedical research applications. SGG inhibits oxidative DNA damage caused by iron (Fe^2+^) in the presence of H_2_O_2_, in a cell free system, which contained plasmid DNA, Fe^2+^ and H_2_O_2_.

### Effect of SGG on ROS

**Figure 9:**
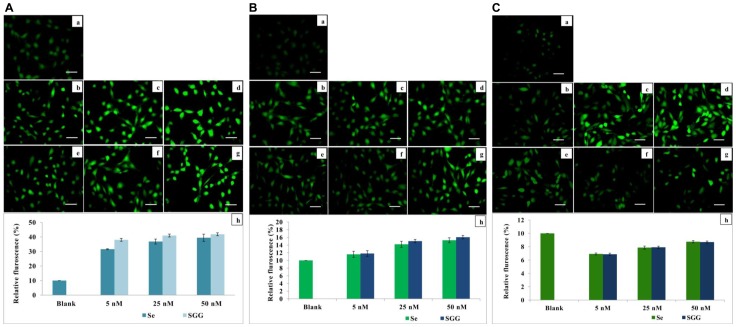
A. Evaluation of ROS with various doses of Se and SGG after 1 Fluorescent microscopic images of H9c2 cells stained with DCFDA (20×). (a) Control cells; (b, c, d) Cells treated with 5, 25 and 50 nM Se, respectively; (e, f, g) Cells treated with 5, 25 and 50 nM of SGG, respectively; (h) The fluorometric analysis supported the microscopic data. It is clear from the fluorescent images that treatment of cells with Se and SGG induce ROS generation i.e., there is a linear increase in fluorescence intensity in dose dependent way (Fig. 9a to g). This indicates ROS generation with Se and SGG in a dose dependent manner. Scale bar corresponds to 100 µm. **B. Evaluation of ROS with various doses of Se and SGG after 6 h**. Fluorescent microscopic images of H9c2 cells stained with DCFDA (20×). (a) Control cells; (b, c, d) Cells treated with 5, 25 and 50 nM Se, respectively; (e, f, g) cells treated with 5, 25 and 50 nM of SGG, respectively; (h) The fluorometric analysis data. It is interesting to note that the magnitude of ROS generation had been decreased in 6 h of incubation compared to 1 h. Scale bar corresponds to 100 µm. **C. Evaluation of ROS with various doses of Se and SGG after 24 h**. Fluorescent microscopic images of H9c2 cells stained with DCFDA (20×). (a) Control cells; (b, c, d) cells treated with 5, 25 and 50 nM Se, respectively; (e, f, g) cells treated with 5, 25 and 50 nM of SGG, respectively; (h) The decrease in ROS production with Se and SGG is the sign of non toxicity of particle to cells for longer duration (24 h). The initial outburst of ROS (Fig. 9A) is most probably due to the self adaptation mechanism of cell to the entry of foreign particle in the form of Se and SGG nanoparticles. Scale bar corresponds to 100 µm.

Induction of oxidative stress is one of the common mechanisms of toxicity of nanoparticles [36]. Oxidative stress occurs when generation of ROS exceed the capacity of antioxidant defense mechanism. It elicits a wide variety of physiological and cellular events including stress, inflammation, DNA damage and apoptosis. In the present study, attempts were made to evaluate ROS generation with various doses of nanoparticle to see whether they induce oxidative stress in H9c2 cells and it was found that treatment with various doses (5, 25 and 50 nM) of Se and SGG caused mild ROS generation (41%) after one hour and gradually reduced to (8%) in a span of 24 h (Fig. 9C). It is interesting to note that the initial outburst of ROS did not cause any morphological alteration or toxicity on cells (Fig. 9A). This temporary elevation of ROS (Fig. 9A) may be due to over reaction of cells for self adaptation to the presence of the foreign material. A mild increase in oxidative stress seen in cells act as a cell signaling mechanism required to trigger several responses to the foreign particle [37]. These results again confirm the suitability of SGG for biomedical application without any adverse effect.

### Alteration in Transmembrane Potential (ΔΨm) of Mitochondria

**Figure 10:**
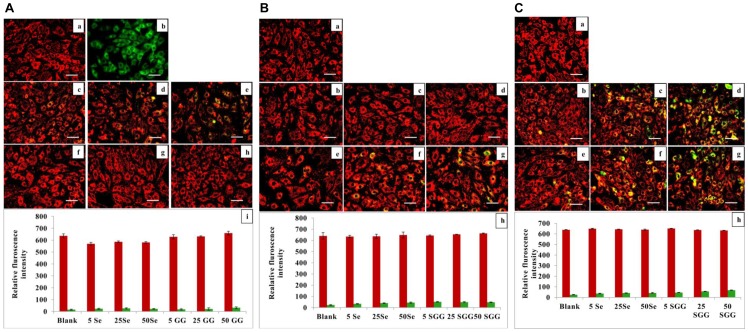
A. Mitochondrial transmembrane potential with Se and SGG after 1 Images of H9c2 cells stained with JC1 for mitochondrial study (20×). (a) Control cells; (b) Positive control valinomycin; (c, d, e) Cells treated with 5, 25 and 50 nM Se, respectively; (f, g, h) Cells treated with 5, 25 and 50 nM SGG, respectively; (i) Relative fluorescence intensity. There was no shift from red to green fluorescence with any groups indicating no change in mitochondrial transmemberane potential. Scale bar corresponds to 100 µm. **B. Mitochondrial transmembrane potential with Se and SGG after 6 h.** Images of H9c2 cells stained with JC1 for mitochondrial study (20×). (a) Control cells; (b, c, d) cells treated with 5, 25 and 50 nM Se, respectively; (e, f, g) Cells treated with 5, 25 and 50 nM SGG, respectively; (h) Relative fluorescence intensity. Here also there is no change of mitochondrial transmembrane potential with any dose of Se and SGG. Scale bar corresponds to 100 µm. **C. Mitochondrial transmembrane potential with Se and SGG after 24 h.** Images of H9c2 cells stained with JC1 for mitochondrial study (20×). (a) Control cells; (b, c, d) Cells treated with 5, 25 and 50 nM Se, respectively; (e, f, g) Cells treated with 5, 25 and 50 nM SGG, respectively; (h) Relative fluorescence intensity. Long duration of exposure of Se and SGG caused some alterations in transmembrane potential of mitochondria at higher dose (50 nM). Scale bar corresponds to 100 µm.

Mitochondria are the vital organelle which play significant role in the physiology of the cells and it is the center target for foreign particles interaction [38]. In this study we verified the effect of various concentration of SGG on mitochondria of H9c2 cells. H9c2 cells are known for the high content of mitochondria to meet its metabolic need. Intact mitochondrion is very much essential for the normal well being of cells as it control many sensitive functions related to energy metabolism. Exposure of H9c2 to Se and SGG for different time duration (1, 6, 24 h) did not cause much alteration in **Δ**Ψm, as measured with JC-1 probe with an average ratio of red:green fluorescence (Fig. 10A, 10B). But high dose (50 nM) caused some alterations in ΔΨm in the case of 24 h of incubation (Fig. 10C).

### Effects of SGG on Cell Cytoskeleton

**Figure 11:**
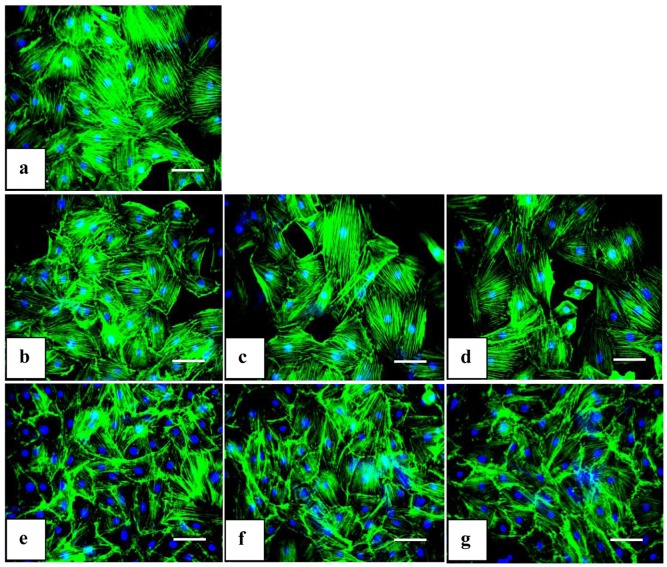
Effect of Se and SGG on cytoskeleton of H9c2 cells. Fluorescent microscopic images of H9c2 cells stained with phalloidin (20×). (a) Control cells; (b, c, d) cells treated with 5, 25 and 50 nM Se, respectively; (e, f, g) cells treated with 5, 25 and 50 nM SGG, respectively. The green colour indicates the cytoskeleton F-actin stained with phalloidin. The blue colour indicates the nucleus stained with DAPI. There were no significant alterations in cytoskeleton with any groups. This reveals clearly that SGG does not cause alteration to cytoskeleton. Scale bar corresponds to 100 µm.

Normal cytoskeleton is essential to keep the morphology and physical structure of the body intact. One possible sign of cellular stress induced by uptake of nanosized materials is alterations to the cytoskeleton network [36]. To check the effect of cellular uptake of nanoparticles on cytoskeleton organization of cardiac myoblast, F-actin component of cytoskeleton was stained using phalloidin. Staining revealed that there was no alteration in structure with Se and SGG treated group. All the groups had an intact filamentous network structure confirming the original structure. Nanoparticles upto 50 nM were safe in holding intact the mesh like architecture of the cells even at 24 h (Fig. 11).

There is not even a single report on SGG nanoparticle and investigation on interaction with cell and this is the first report in this regard. But there are reports of preparation of Se nanoparticles with hyperbranched polysaccharide in water [39]. However, it was not prepared for any biological applications. In addition Se nanoparticles with spirulina polysaccharide had been prepared by some groups [30] for evaluation of its anticancer activities and uptake of Se nanoparticle by cells. They reported enhanced cytotoxicity and uptake by melanocytes. In the case of guar gum, there are reports on silver nanoparticle preparation using polyacrylamide/guar gum graft copolymers for some chemical purpose [40]. Green synthesis had also been exploited for preparation of biopolymer (GG) silver nanoparticle composite for application in optical sensor for ammonia detection [41]. Overall results clearly reveal SGG prepared in this study exhibit potent therapeutic potential with respect to oxidative stress, apoptosis, DNA protection, mitochondrial membrane potential and cytoskeleton. Moreover the cytotoxicity was minimum with SGG which is a great advantage of this particle for biomedical applications.

## Conclusion

The present study demonstrated that SGG can be successfully prepared under mild conditions via, nanoprecipitation method. Physicochemical properties, such as particle size, TEM, XRD data confirmed the nanoscale structure of the prepared material and proper incorporation of selenium to guar gum nanoparticles. Elaborate investigations on the interaction of nanoparticle on biological system had been conducted on H9c2 and confirmed the safety of nanoparticle on biological system (H9c2). On the basis of these results we assume that that SGG is an ideal nanomaterial for further research with promising therapeutic properties especially for cardiac problems.
